# Regulatory effects of *Lactobacillus plantarum* HY7714 on skin health by improving intestinal condition

**DOI:** 10.1371/journal.pone.0231268

**Published:** 2020-04-10

**Authors:** Bora Nam, Soo A. Kim, Soo Dong Park, Hyeon Ji Kim, Ji Soo Kim, Chu Hyun Bae, Joo Yun Kim, Woo Nam, Jung Lyoul Lee, Jae Hun Sim

**Affiliations:** R&BD Center, Korea Yakult Co. Ltd., Yongin, Republic of Korea; Kyungpook National University, REPUBLIC OF KOREA

## Abstract

Despite increasing research on the gut-skin axis, there is a lack of comprehensive studies on the improvement of skin health through the regulation of the intestinal condition in humans. In this study, we investigated the benefits of *Lactobacillus plantarum* HY7714 (HY7714) consumption on skin health through its modulatory effects on the intestine and ensuing immune responses. HY7714 consumption led to differences in bacterial abundances from phylum to genus level, including increases in Actinobacteria followed by *Bifidobacterium* and a decrease in Proteobacteria. Additionally, HY7714 significantly ameliorated inflammation by reducing matrix metallopeptidases (MMP-2 and MMP-9), zonulin, and calprotectin in plasma, all of which are related to skin and intestinal permeability. Furthermore, RNA-seq analysis revealed its efficacy at restoring the integrity of the gut barrier by regulating gene expression associated with the extracellular matrix and immunity. This was evident by the upregulation of IGFBP5, SERPINE1, EFEMP1, COL6A3, and SEMA3B and downregulation of MT2A, MT1E, MT1X, MT1G, and MT1F between TNF- α and TNF- α plus HY7714 treated Caco-2 cells. These results propose the potential mechanistic role of HY7714 on skin health by the regulation of the gut condition.

## Introduction

The relationship between the gut in terms of intestinal microflora and skin health, termed the “gut-skin axis,” via the immune system is a well-known theory.[1–3] Increasing studies show that probiotics can regulate the skin condition, providing physiological evidence of their potentially therapeutic characteristics. *Lactobacillus reuteri* ATCC 6475 fed mice had thicker skin and more lustrous fur than their control counterparts, due to the presence of anti-inflammatory cytokine interleukin-10.[4] Similarly in a human study, supplementation of *L*. *plantarum* CJLP133 contributed to the alleviation of atopic dermatitis and decreased eosinophil counts [5] whereas *L*. *paracasei* NCC 2461 consumption reduced skin sensitivity and transepidermal water loss (TEWL).[6]

*Lactobacillus plantarum* HY7714 (HY7714) is one of the probiotics permitted by the Korea Food and Drug Administration (KFDA) because of its benefits of skin hydration and UV protection. It effectively hydrates the epidermis via the regulation of serine palmitoyltransferase (SPT) and ceramidase mRNA levels.[7] It also improves photoaging—skin damage due to UV light; in HY7714 treated mice, UVB-induced matrix metalloproteinase (MMP) -13, -2 and -9 was inhibited subsequently decreasing the number, depth, and area of skin wrinkles.[8] Furthermore, in a clinical trial involving 110 healthy women with dry skin and wrinkles, HY7714 consumption improved skin hydration and reduced wrinkle depth.[9]

Probiotics have also been reported to improve the integrity and immunomodulation of the gut epithelial barrier [10,11] evident in recent studies on HY7714, which showed its restorative effects in defects of tight junctions in human intestinal Caco-2 cells.[12] HY7714 treatment recovered a decrease in tight junction proteins (zonula occludens-1, occludin, and claudin-1) induced by TNF-α, and attenuated myosin light chain kinase (MLCK) expression thought to disrupt the cytoskeletal structure of the tight junction, and pro-inflammatory cytokines thought to induce TJ permeability.[12] From these findings, we extrapolated that HY7714 could improve the skin condition by stabilizing intestinal permeability.

To reinforce the effects of HY7714 on the intestinal wall (barrier), we investigated the transcriptome sequencing of differentially expressed genes (DEGs) between TNF-α and TNF-α plus HY7714 treated Caco-2 cells and categorized our results according to GO enrichment analysis categorized under biological process, cellular component, and molecular function ontology.

Furthermore, many studies refer to the relationship between the gut and skin health via immunological modifications,[13–16]; however, the impact of HY7714 on intestinal microbiota and relative inflammatory biomarkers in humans is not yet understood. In this way, we recruited healthy volunteers and supplemented 1 × 10^10^ CFU of HY7714 for 8 wk to confirm its regulatory effects on gut microbiota and biomarkers related with inflammation, skin conditions, and intestinal permeability.

## Materials and methods

### Preparation of HY7714 for in vitro assays

HY7714 was inoculated in de Man–Rogosa–Sharpe (BD, USA) broth, cultured at 37°C for 20 h, harvested by 1,500 × g centrifugation for 10 min, washed two times with sterile phosphate-buffered saline (PBS), and resuspended to a final concentration of 1 × 10^10^ CFU/ml. Afterwards, it was heat treated at 100°C for 15 min and stored at –20°C until ready for further assay.

### Cell culture

Caco-2 [17] human colorectal adenocarcinoma epithelial cells were purchased from the Korean Cell Line Bank (Seoul, Korea KCLB 30037.1) and cultured in Eagle's Minimum Essential Medium supplemented with 20% fetal bovine serum in a humidified atmosphere of 5% CO_2_ at 37°C. Cells were harvested with trypsin-EDTA solution, placed in a 6-well plate (1 × 10^5^ cells/well), and grown for 21 d to reach differentiation. Growth media was renewed 1 to 2 times per week.

### Treatment of HY7714 on Caco-2 cells

The fully differentiated Coco-2 cells were serum deprived overnight and treated with 100 ng/ml of TNF-α (Sigma-Aldrich Co., St. Louis, MO, USA) and 1 × 10^8^ CFU/ml of heat-treated *L*. *plantarum* HY7714 for 24 h. Cells were divided to 3 groups; TNF-α only, TNF-α plus HY7714 treatment, and no treatment (control) and stored in TRIzol® solution (Thermo Fisher Scientific, USA) at -80°C until ready for total RNA isolation.

### RNA-sequencing and bioinformatics analysis

RNA extraction and subsequent sequencing was conducted at the Teragenetex Bio Institute (Suwon, Korea). Total RNA was extracted from TNF-α and HY7714 treated cells and their concentration and quality were measured. The mRNA sequencing library was constructed using the TrueSeq Stranded mRNA Preparation Kit (Illumina, San Diego, USA) and used for cluster generation and NGS with the NovaSeq Sequencing platform (Illumina, San Diego, USA) following the manufacturer’s instructions. The transcriptome sequencing was performed using the Ribosomal Database Project (RDP) and National Center for Biotechnology Information (NCBI) database. Gene Ontology (GO) enrichment analysis was conducted on the database for Annotation, Visualization, and Integrated Discovery DAVID Bioinformatics Resources 6.8 (https://david.ncifcrf.gov/home.jsp) based on the differentially expressed genes.

### Participants and experimental design

**Fig 1:**
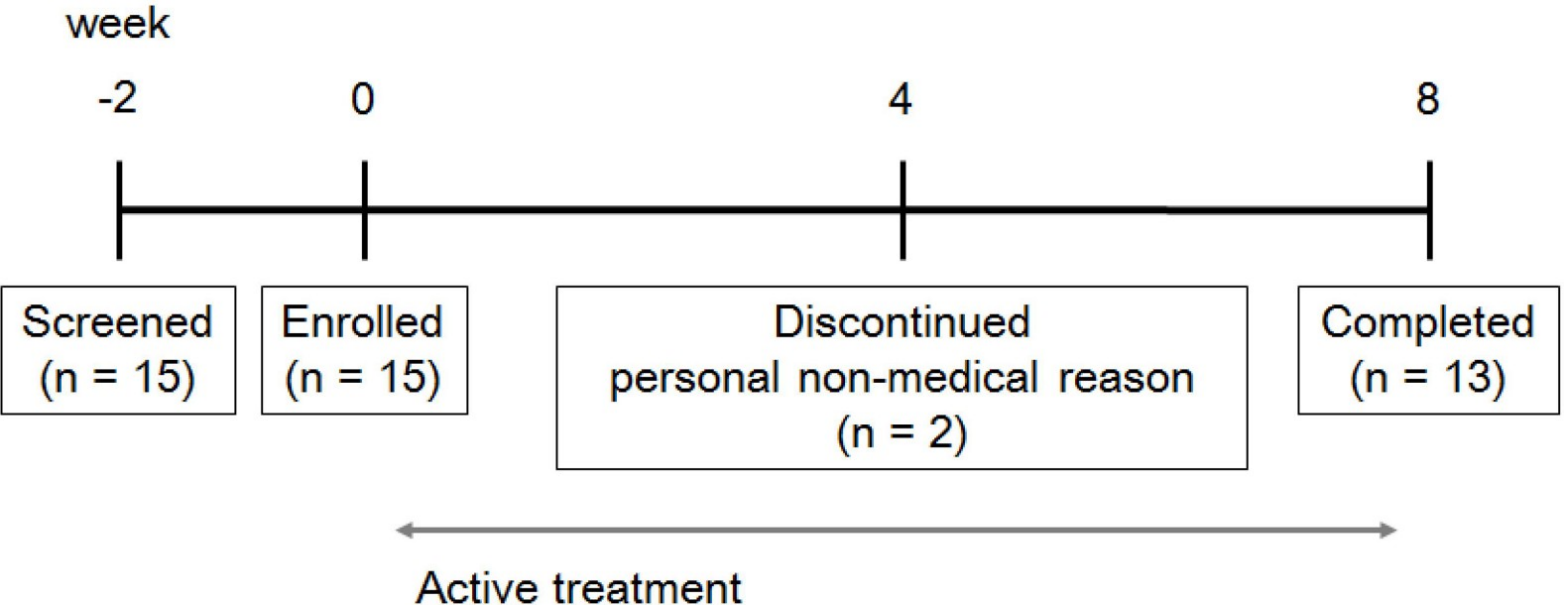
Flow chart of the study.

We recruited healthy female volunteers aged 23 to 67 y old by a poster at the Vievis Namuh Hospital (Seoul, Korea) from August to September 2019. Participants were selected through the survey about inclusion and exclusion criteria. All the subjects were informed of the purpose and the expectations of the study and wrote the consent form to be enrolled in the study. Table 1 reports demographic features of subjects. In total, 15 female volunteers were enrolled and 13 of them completed the study; two subjects withdrew due to personal non-medical reason (**Fig 1**). This study was conducted in accordance with the Institutional Review Board of Vievis Namuh Hospital (Korea; VNIRB IRB No.201901) and approved by the Ethical Committee of the VNIRB (Seoul, Korea).

All the enrolled subjects were given a 450 mg capsule containing 1 × 10^10^ CFU of HY7714 daily for 8 wk. *Lactobacillus plantarum* HY7714 is an approved probiotic that is effective at skin hydration and UV protection. It was obtained from a Korea Yakult Pyeongtaek probiotics plant (Pyeongtaek, Korea) and its lot number is 19-7714-005.

**Table 1:** Demographic characteristics of subjects.

FAS group (n = 15)	Mean ± SD	Median	Range
**Age (years)**	44.3±16.5	41	23~67
**Height (cm)**	161.1±4.6	160.3	153.1~168.9
**Weight (kg)**	56.5±6.8	53.5	49~71

### Exclusion criteria

1. Pregnancy, possible pregnancy or lactating
2. Currently or previously diagnosed with gastrointestinal diseases, such as Crohn’s and celiac disease, ulcerative colitis, and malignant tumors of the colon
3. Intake of probiotics or antibiotics consistently in a week
4. Participation in a similar previous study within 3 months
5. The presence of a disease that may interfere with the study, such as cardiac, renal, liver, hyperthyroid, cerebrovascular, gall bladder, or gastrointestinal disorders, and podagral
6. Active skin diseases
7. The presence of a chronic diseases, such as asthma, diabetes, and high blood pressure
8. The presence of a mental illness, such as schizophrenia, alcoholism, or drug abuse
9. Diagnosed inappropriate for the study due to conditions not stated above
10. History of adverse effects of probiotics.

### Fecal sampling and total fecal DNA extraction

Fecal samples were collected three times (0, 4, and 8 wk) from all participants during the 8 wk study. Sample collection kits were provided with a plastic container, packed within an insulated bag, and chilled with frozen gel packs. The fecal samples were then stored at –80°C until ready for fecal DNA extraction. Total fecal DNA samples were extracted using QIAamp DNA Stool mini kit (Qiagen, USA).

### 16S rRNA gene PCR for NGS analysis

Extracted total fecal DNA samples were diluted for 16S rRNA gene PCR and gene amplification was conducted for preparation of fecal DNA sequencing templates. The V3-V4 region of 16S rRNA sequence was screened using the 341F (341 forward primer, 5´-CCT ACG GGN GGC WGC AG-3´) and 805R primers (805 reverse primer, 5´-GAC TAC HVG GGT ATC TAA TCC-3´). For PCR amplification, the PCR mixture contained fecal DNA sample, 2× Kapa Hifi Hotstart ready mix (Kapa Biosystems, USA), and the forward and reverse primers. PCR products were quantified and used as 16S rRNA amplicons for NGS sequencing.

### Bioinformatics analysis of metagenomics

The raw data was analyzed for intestinal microbial composition analysis. The NGS analysis of raw sequence data was collected using Illumina MeSeq (Theragen Etex, Suwon, Korea). Paired-end sequences were assembled and quality control (QC) retained sequences with 300 bp length were then used for operational taxonomic unit (OTU) clustering based on the 16S rRNA sequences database. Taxonomic assignment was done using the NCBI database and RDP (Ribosomal Database Project) to analyze the gut microbiome composition of each sample. The differential abundance of taxa before and after supplementation of HY7714 was determined at the OTU level and relative abundance comparisons at the genus, family, and phylum levels were performed on normalized data using a Wilcoxon signed-rank test. Alpha diversity was calculated using the following equation [18]:

$$Shannon\;index,\;H'=\sum_{i=1}^{S}\left(p_{i}ln{(p}_{i})\right).$$

### Biomarker changes in plasma

The blood samples were collected at the Hospital at 0, 4, and 8 wk after HY7714 consumption and stored -80°C until ready for further analysis. The levels of zonulin, calprotectin (R&D systems), and MMP-2, and MMP-9 (CUSABIO, Houston, TX, USA) were measured using enzyme-linked immunosorbent assay (ELISA) kits according to the manufacturer’s instructions.

### Statistical analysis

All data were presented as the mean ± standard deviation (SD). To compare between groups, we performed a paired Student’s t-test or two-tailed Wilcoxon signed-rank test in accordance with the variable distribution. A p value of < 0.05 or < 0.01 was considered statistically significant.

## Results

### RNA-seq and functional gene ontology analysis

To explore HY7714 mediated transcriptional changes, we conducted RNA-seq analyses between TNF-α and TNF-α plus HY7714 treated Caco-2 cells. An average of 1.2 hundred million raw reads were generated and 99.4% of them were used after quality filtering. These were mapped to the human reference genome with 82.6% mapped uniquely. Subsequently, 23,043 expressed genes were identified using cufflinks; 189 genes were identified as DEGs between TNF-α and TNF-α plus HY7714 treated cells based on a p value < 0.05 and fold change > 2. We generated a volcano plot to visualize transcriptomic differences between TNF-α and TNF-α plus HY7714, based on a log2 fold change.

**Fig 2:**
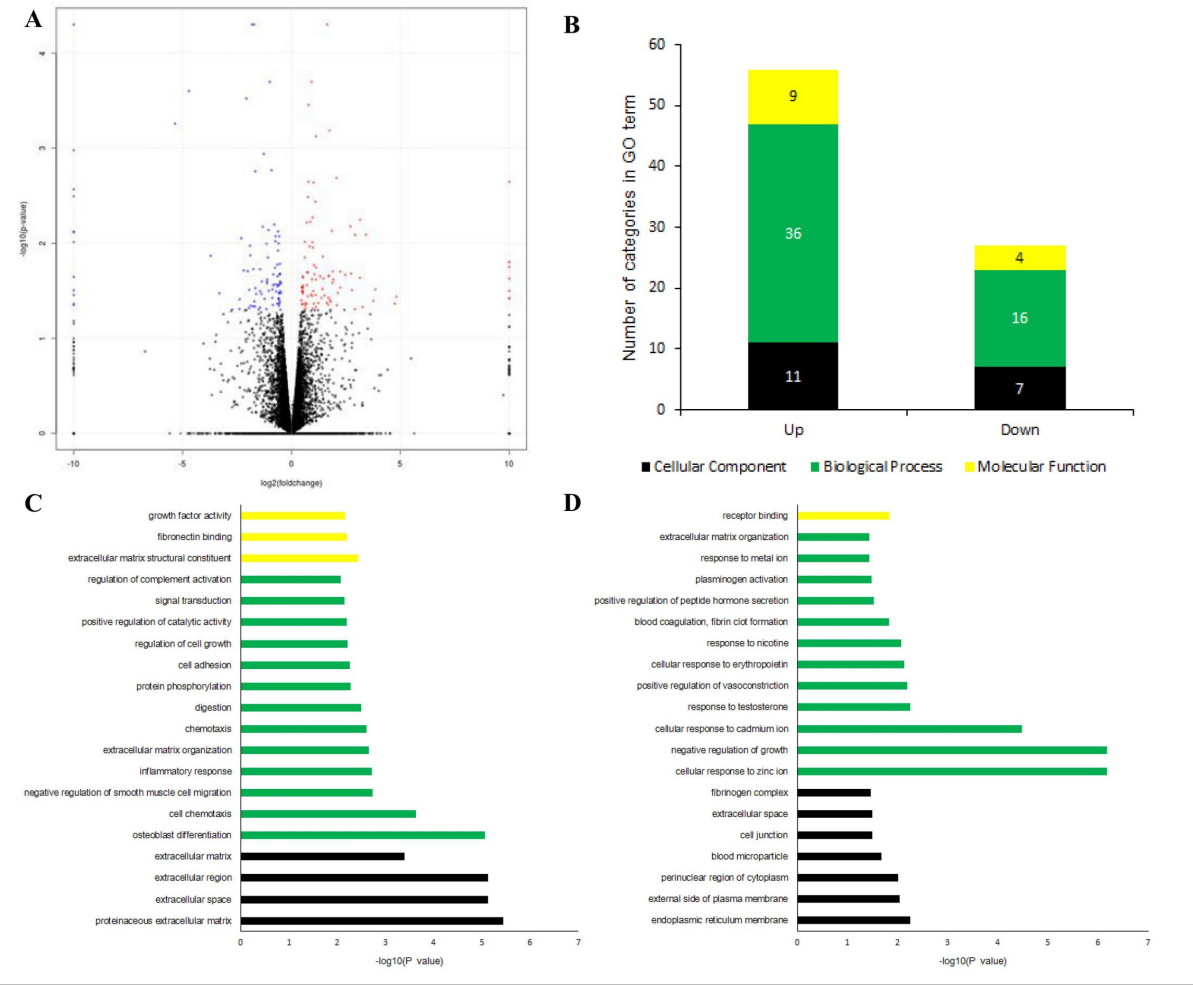
RNA-seq data and gene ontology analysis of TNF-α and TNF-α plus HY7714 treated Caco-2 cell. (A) Volcano plot between TNF-α and TNF-α plus HY7714 treatment. FC (fold change) > 2 was accepted as differentially expressed; red: up-regulated and blue: down-regulated. DEGs are analyzed by GO terms. The number of categories in each GO (B), and the top 20 GO terms up regulated (C) and down regulated (D). Yellow, green, and black indicate genes that belongs to the molecular function, biological process, and cellular component categories. Terms were considered significant at p < 0.05.

Of these, 99 genes were up-regulated and 90 genes were down-regulated as shown in red and blue dots in **Fig 2A**. Next, we categorized differentially regulated genes by the HY7714 treatment based on the TNF-α treatment according to GO analysis based on the biological process, cellular component, and molecular function ontology functions (**Fig 2B**). The top 20 categories significantly enriched (p < 0.05) within ontology were arranged to–log (p value) order in each GO (**Fig 2C and 2D**). The 99 up-regulated genes associated with the proteinaceous extracellular matrix (GO: 0005578), extracellular space (GO: 0005615), and extracellular region (GO: 0005576) were highly enriched in the cellular component category. Genes involved in osteoblast differentiation (GO: 0001649), cell chemotaxis (GO: 0060326), and negative regulation of smooth muscle cell migration (GO: 0014912) were most frequent in the biological process category. The molecular function category included genes highly involved in extracellular matrix structural constituents (GO: 0005201), fibronectin binding (GO: 0001968), and growth factor activity (GO: 0008083).

Of the 90 down-regulated genes, those involved in the endoplasmic reticulum membrane (GO: 0005789), external side of plasma membrane (GO: 0009897), and perinuclear region of cytoplasm (GO: 0048471) were most frequently found in the cellular component category. In the biological processes category, cellular responses to zinc ion (GO: 0071294) and negative regulation of growth (GO: 0045926) were equally most identified followed by cellular response to cadmium ion (GO: 0071276). Receptor binding (GO: 0005102) alone had downregulated genes in the molecular function category. Insulin like growth factor binding protein 5 (IGFBP5), Plasminogen activator inhibitor-1 (SERPINE1), EGF containing fibulin extracellular matrix protein 1 (EFEMP1), and collagen type VI alpha 3 chain (COL6A3), semaphorin 3B (SEMA3B) were highly up-regulated after HY7714 treatment. These mostly belong to the proteinaceous extracellular matrix, extracellular space, and extracellular region categories of cellular component ontology. The top down-regulated genes, metallothionein 2A (MT2A), metallothionein 1E (MT1E), metallothionein 1X (MT1X), metallothionein 1G (MT1G), and metallothionein 1F (MT1F) belonged to the cellular response to zinc ion and negative regulation of growth categories of biological processes.

### Study design and demographic features of subjects

Participants were recruited from Vievis Namuh Hospital (Seoul, Korea) based on aforementioned criteria (see Methods section). Table 1 reports demographic features of subjects. In total, 15 female volunteers were enrolled and 13 of them completed the study; two subjects withdrew due to personal non-medical reason.

### Overview and diversity analysis of gut microbiota

**Fig 3:**
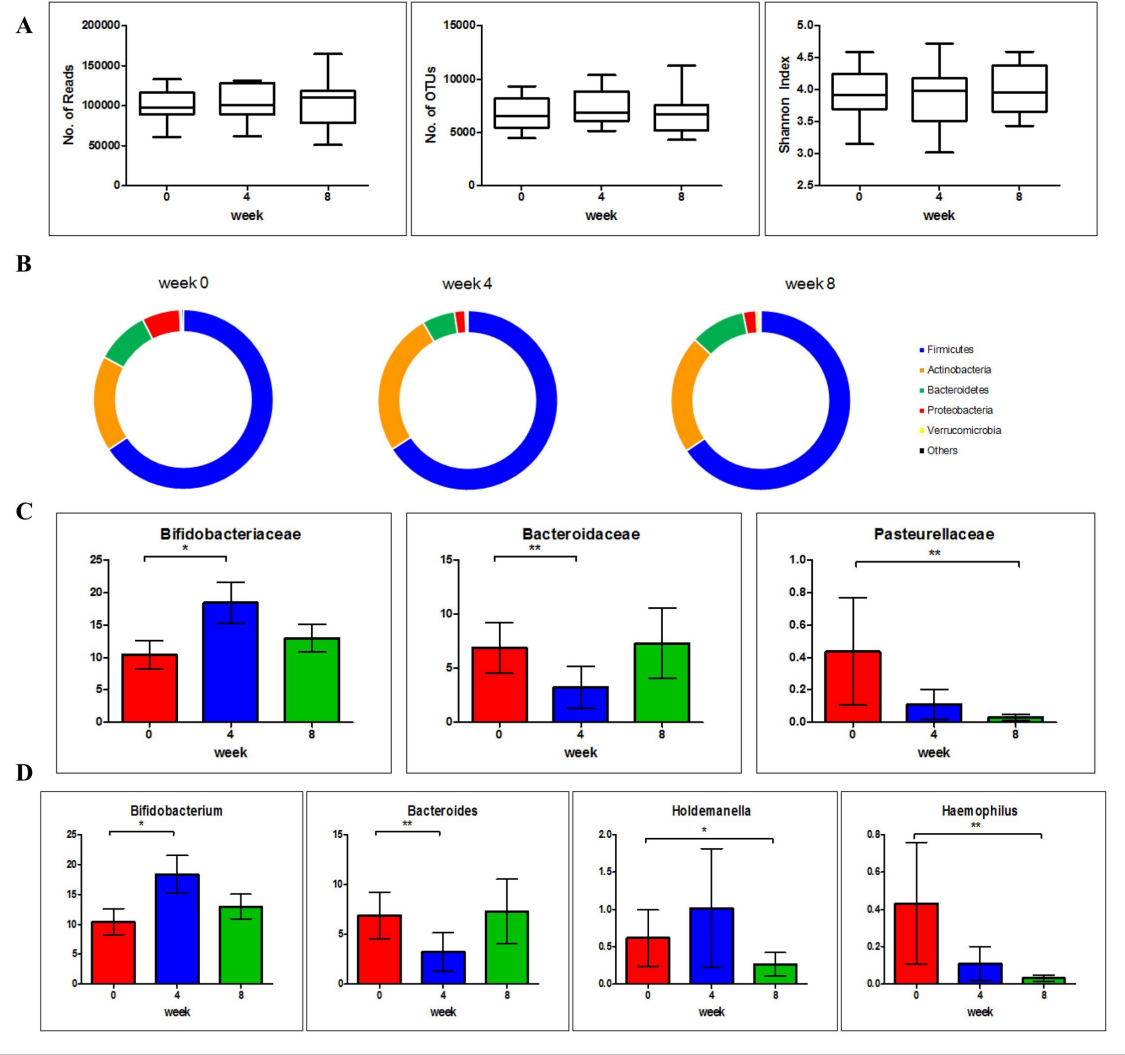
Gut microbial composition in PPS groups before and after HY7714 consumption at 4 and 8 wk. Sequence summary in fecal samples; the number of reads, operational taxonomic units (OTUs), and Shannon index of alpha diversity (A). Relative abundances of gut microbial composition at the phylum (B), family (C) and genus levels (D). Data are analyzed using the Wilcoxon signed-rank test. Results are expressed as the Mean ± SEM. Asterisks (* and **) indicate a significant difference (p < 0.05 and p < 0.01, respectively) in comparison to the control. PPS: per protocol set.

To analyze the changes in gut microbiota caused by HY7714 consumption, we performed a metagenome sequencing by targeting the V3-V4 region of the 16S rRNA gene with Illumina Miseq. We obtained an average of 102,628 ± 25,665 reads per participant and then clustered operational taxonomic units (OTUs) using the NCBI and RDP database at 97% identity (**Fig 3A**). We recorded an average of 6,931 OTUs per sample (Fig 3). The alpha diversity was measured using the Shannon index. The Shannon index at 0 wk was 3.9399 ± 0.3676 (ranged from 3.1515 to 4.5858) and decreased at wk 4 to 3.9010 ± 0.4878 (ranged from 3.0170 to 4.7192), then increased at wk 8 to 3.9981 ± 0.3938 (ranged from 3.4320 to 4.5934).

### Intestinal microbial composition of HY7714 supplemented participants

Across all subjects, the dominant phyla were Firmicutes and Actinobacteria, which made up 65.71 ± 0.12% and 21.47 ± 4.29%, respectively of the total abundance, with contributions from Bacteroidetes 8.50 ± 2.29%, Proteobacteria 3.71 ± 2.71%, and Verrucomicrobia 0.38 ± 0.16%. The dominant bacterial families were Lachnospiraceae, Ruminococcaceae, Bifidobacteriaceae, Lactobacillaceae, Coriobacteriaceae, Erysipelotrichaceae, and Bacteroidaceae at 16.06, 15.06, 14.31, 8.74, 6.67, 5.47 and 5.10%, respectively.

Between groups, there were significant differences in Actinobacteria and Bacteroidetes at the phylum level after 4 wk of HY7714 supplementation. Relative abundances of Actinobacteria and Verrucomicrobia increased, but Proteobacteria decreased at the phylum level of microbial composition. Interestingly, Bacteroidetes decreased after 4 wk and then returned to a basal level at 8 wk, and this was influenced by changes in Bacteroidaceae and Bacteroides at the family and genus levels, respectively.

The increase in Actinobacteria was evident by elevated levels of Bifidobacteriaceae and *Bifidobacterium* at the family and genus levels, respectively. Within Proteobacteria, the family Pasteurellaceae and genus *Haemophilus* significantly decreased in a time-dependent manner. *Haemophilus* spp. are commensal pathogenic organisms, including *H*. *influenza*. Similarly, the genus *Holdemanella* was significantly lower at wk 8 than wk 0. According to a previous study, the genus *Holdemanella* was more abundant in constipated patients than healthy individuals [19].

Overall, there were prominent changes in Actinobacteria, Bacteroidetes, and Proteobacteria at wk 4 which were, to some extent, reversed by wk 8. In response to *Lactobacillus* supplementation, *Bifidobacterium* increased and pathogenic bacteria, such as *Holdemanella* and *Haemophilus*, decreased. This is an indication of the beneficial effects of HY7714 consumption in the modulation of intestinal microbiota.

### HY7714 decreased the level of biomarkers related with inflammation

Zonulin in plasma decreased by 26.92% at wk 4 and 8. Calprotectin concentrations also lowered after 4 wk of HY7714 consumption by 29.17% (p < 0.05) and this level was maintained to 8 wk. Other types of cytokines, such as TNF-α, IL-6, IL-10, TSLP, and eotaxin tended to decrease after HY7714 consumption, but not significantly (**S2 Fig**).

**Fig 4:**
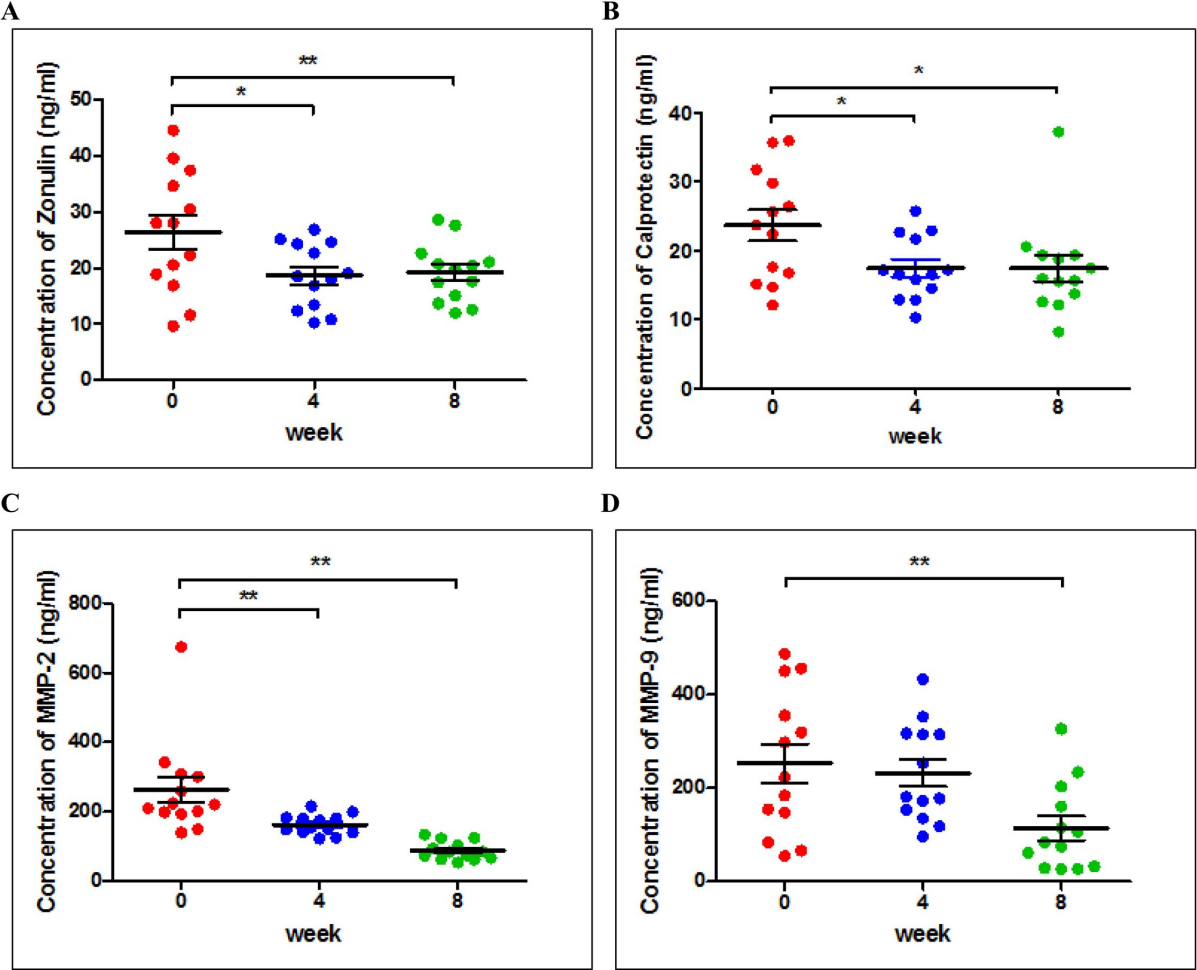
Comparison of biomarker concentrations in plasma. Changes in Zonulin(A), Calprotectin(B), MMP-2(C) and MMP-9(D) levels in plasma before and after HY7714 consumption over 8 wk. Data are analyzed using the Wilcoxon signed-rank test. Results are expressed as Mean ± SEM. Asterisks (* and **) indicate a significant difference (p < 0.05 and p < 0.01, respectively) compared to the control. Each dot represents an individual. PPS: per protocol set.

The plasma concentration of MMP-2 and MMP-9 of subjects decreased over time (**Fig 4D**). MMP-2 levels decreased by 38.02% and 66.54% at wk 4 and 8, respectively (p < 0.01) whereas there was a 54.76% decrease in MMP-9 at wk 8 (p < 0.01).

## Discussion

Despite overwhelming physiological evidence of the relationship between the gut and skin conditions,[1–3] there is no comprehensive study on the intestinal microbiome, relative inflammatory biomarkers, and changes in the skin health of individuals taking probiotic supplements. Previously we demonstrated the anti-aging properties of HY7714 consumption, such as hydration, elasticity and wrinkles, on the skin [9] and confirmed its effects on gut microflora composition and immunomodulation in fecal and plasma samples of participants before and after HY7714 consumption. We observed significant taxonomic differences in microbial composition and decreases in inflammatory biomarkers. Furthermore, based on RNA seq analysis we concluded that this probiotic had a regulatory impact on the intestine other than restoring defects of tight junctions in Caco-2 cells. [12] We found differing gene expression levels between TNF-α and TNF-α plus HY7714 treatment groups in the cellular component, biological processes, and molecular function ontology categories. We predicted differences in genes associated with intestinal integrity. Highly up-regulated genes mostly belonged to the cellular component category.

IGFBP-5 binds to endothelial cell monolayers by its cysteine residues [20] and could act in tissue remodeling. It also stimulates collagen production and upregulates extracellular matrix genes [21] and is reported to inhibit phosphorylation of ERK1/2, and p38-MAPK kinases.[22] EFEMP1 encodes glycoproteins composing the extracellular matrix component.[23] It also regulate apoptosis of carcinoma cells mediated by SEMA3B,[24] which solely induces apoptosis of tumor cells and is therefore a known tumor suppressor [25,26]. COL6A3 works to bind extracellular matrix proteins suggesting an interaction with collagen in organizing matrix components.[27] SERPINE1 functions in tissue repair and regulates plasmin formation, which degrades extracellular matrices that may favor cancer cell invasion [28,29].

Due to the up-regulation of these genes after HY7714 treatments, we proposed that HY7714 repairs defects on the extracellular matrix in intestinal cells derived by TNF-α and prevents penetration of inflammatory factors.

Metallothioneins, including MT2A, MT1E, MT1X, MT1G, and MT1F, is a cysteine-rich family with the capacity to bind to heavy metals, such as zinc, copper, and cadmium and therefore must play a role in zinc homeostasis [30]. Zinc dysregulation could result in immune-pathologies, gastrointestinal dysregulation, and cancer due to the formation of reactive oxygen species.[31,32]

Furthermore, metallothioneins and zinc regulate the activation of NF-kB[31], which increase tight junction (TJ) permeability by suppressing the expression of tight junction proteins [33] and enhance the disassembly of actin-myosin complexes via the up-regulating of MLC phosphorylation [34]. The imbalance of metallothionein expression can be observed in various diseases and MT2A, MT1G, and MT1X were up regulated in response to TNF-α treatment and downregulated after HY7714 treatment. These results parallel those of a previous study on the therapeutic function of HY7714 to TJ defects derived by TNF-α.[12]

*Bifidobacterium* is one of the probiotics, which exerts many benefits to the host. It displays anticancer activities [35], and can be used to treat disorders, such as diarrhea and inflammatory bowel disease [10], and decreases intestinal permeability by increasing stabilization and regulating the expression of tight junction proteins [36,37]. Intestinal permeability increases with inflammation and inflammation factors decrease with *Bifidobacterium* treatment [31,38].

It is intriguing that *Lactobacillus* consumption increased *Bifidobacterium* in the intestine. Probiotics involved in physiological functions that alter gut microbiota composition and specific bacteria promote the growth of other probiotics and inhibit pathogenic bacteria. [39] In this study, HY7714 contributed to the prominence of *Bifidobacterium* in the gut microbiota and this may have been induced by one of the characteristics of probiotics that modulate gut microbiota positively.

HY7714 consumption also decreased Proteobacteria phyla, influenced by bacteria of the Pasteurellaceae family and *Haemophilus* genus. Healthy humans have a low abundance of Proteobacteria thus, it is used as a diagnostic biomarker for dysbiosis and disease. [40] *Haemophilus* is known to include some species that increase and are involved in the pathology of multiple sclerosis.[41] *Holdemanella*, a genus of the Firmicutes phyla, also decreased in response to HY7714 consumption; its levels are associated with constipation, an unbalanced lipid profile, and chronic kidney disease. [19,42,43]

Intestinal microbiota interact with other microbes and modulate physiologic and metabolic processes. They provide specific functions and maintain gut homeostasis. [10,39] The change in composition in gut microflora began after 4 wk of HY7714 consumption and was restored, to some extent, at wk 8; this recovery may be led by gut homeostasis of commensal microflora. We are limited by the lack of longer term results; regardless, there was a tendency for beneficial bacteria to increase and harmful bacteria to significantly decrease.

Matrix metallopeptidases (MMPs) are zinc containing endopeptidases, which degrade extracellular matrix proteins thought to contribute to skin aging [44]. MMP-2 and MMP-9 are collagenases capable of degrading triple helical fibrillar collagens [45]. Their expression is increased under conditions of gut dysbiosis with high abundance of certain pathogens and for patients having inflammation related diseases, such as colorectal cancer and inflammatory bowel disease [46,47]. Its levels significantly dropped after HY7714 supplementation suggesting it may attenuate inflammatory effects and collagen destruction in skin.

Zonulin is a protein that activates intestinal permeability by modulating tight junctions [48] whereas calprotectin is a complex of mammalian proteins that are secreted during inflammation, so it could serve to detect diseases, such as inflammatory bowel diseases and rheumatoid arthritis [49]. It sequestrates transition metal ions, such as manganese and zinc via chelation [50]. Both zonulin and calprotectin levels decreased after HY7714 consumption, suggesting that it plays a role in the improvement of intestinal integrity and has anti-inflammatory effects.

HY7714 improves the gut microbiome community and biomarker levels relevant to anti-inflammation. Its effects could also be extrapolated to skin health through the regulation of the gut and relative immune reactions based on the gut-skin axis mechanism. However, future longer term and more in-depth studies need to be conducted to make definitive conclusions on the role of this probiotic and its relationship with the gut-skin axis.

## Supporting information

S1 Fig Changes of skin condition after 8 wk of HY7714 consumption. Changes in skin water content (A) and transepidermal water loss (B) measured at three areas (face, forearm, and hand) every 4 wk for 8 wk. Data are analyzed using the Wilcoxon signed-rank test. Results are expressed as Mean ± SEM. Asterisks (* and **) indicate a significant difference (p < 0.05 and p < 0.01, respectively) compared to the baseline.
(DOCX)

S2 Fig Changes of cytokine levels in plasma after 8 wk of HY7714 consumption. Changes in concentration of TNF-α (A), IL-6 (B), IL-10 (C), TSLP (D), and eotaxin (E) measured every 4 wk for 8 wk. Results are expressed as Mean ± SEM.
(DOCX)

S1 Table Gut microbial composition before and after HY7714 consumption. Relative abundances of gut microbial composition at the family (A), and genus levels (B).
(DOCX)

S2 Table Biomarker concentrations in plasma before and after HY7714 consumption. Zonulin (A), Calprotectin (B), MMP-2 (C), and MMP-9 (D).
(DOCX)
