# Supplementary material for: Regulatory effects of Lactobacillus plantarum HY7714 on skin health by improving intestinal condition
Source: PLoS One. 2020 Apr 10;15(4):e0231268. doi: 10.1371/journal.pone.0231268 (PMC7147770; doi:10.1371/journal.pone.0231268)
Supplement: S1 Fig — Changes in skin water content (A) and transepidermal water loss (B) measured at three areas (face, forearm, and hand) every 4 wk for 8 wk. Data are analyzed using the Wilcoxon signed-rank test. Results are expressed as Mean ± SEM. Asterisks (* and **) indicate a significant difference (p < 0.05 and p < 0.01, respectively) compared to the baseline. (DOCX) [file pone.0231268.s001.docx]

A


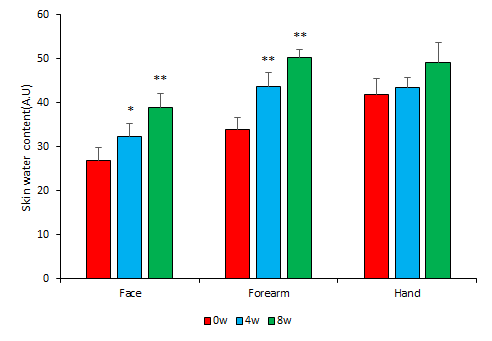


B


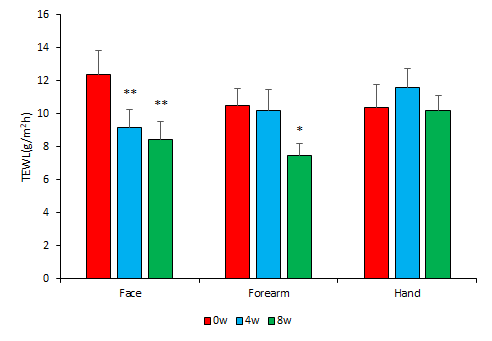


**S1 Fig. Changes of skin condition after 8 wk of HY7714 consumption.**

Changes in skin water content (A) and transepidermal water loss (B) measured at three areas (face, forearm, and hand) every 4 wk for 8 wk. Data are analyzed using the Wilcoxon signed-rank test. Results are expressed as Mean ± SEM. Asterisks (* and **) indicate a significant difference (p < 0.05 and p < 0.01, respectively) compared to the baseline.
