# Supplementary material for: Regulatory effects of Lactobacillus plantarum HY7714 on skin health by improving intestinal condition
Source: PLoS One. 2020 Apr 10;15(4):e0231268. doi: 10.1371/journal.pone.0231268 (PMC7147770; doi:10.1371/journal.pone.0231268)
Supplement: S2 Fig — Changes in concentration of TNF-α (A), IL-6 (B), IL-10 (C), TSLP (D), and eotaxin (E) measured every 4 wk for 8 wk. Results are expressed as Mean ± SEM. (DOCX) [file pone.0231268.s002.docx]

A B


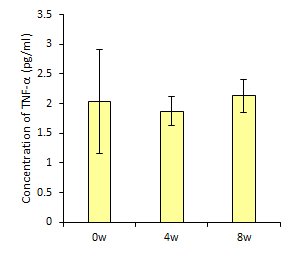

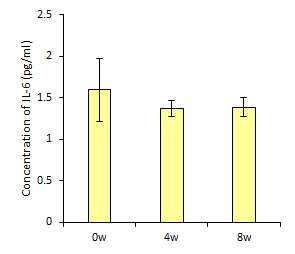


C D


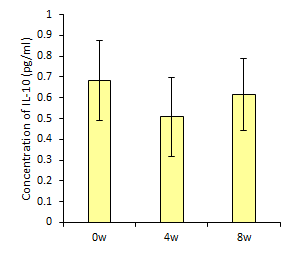

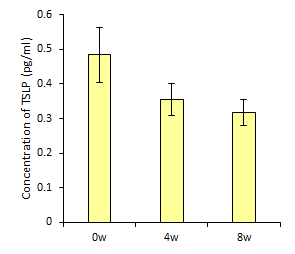


E


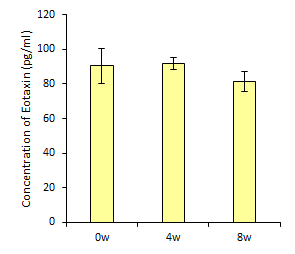


**S2 Fig. Changes of cytokine levels in plasma after 8 wk of HY7714 consumption.**

Changes in concentration of TNF-α (A), IL-6 (B), IL-10 (C), TSLP (D), and eotaxin (E) measured every 4 wk for 8 wk. Results are expressed as Mean ± SEM.
