# Supplementary material for: Regulatory effects of Lactobacillus plantarum HY7714 on skin health by improving intestinal condition
Source: PLoS One. 2020 Apr 10;15(4):e0231268. doi: 10.1371/journal.pone.0231268 (PMC7147770; doi:10.1371/journal.pone.0231268)
Supplement: S1 Table — Relative abundances of gut microbial composition at the family (A), and genus levels (B). (DOCX) [file pone.0231268.s003.docx]

**S1 Table. Gut microbial composition before and after HY7714 consumption.**

Relative abundances of gut microbial composition at the family (A), and genus levels (B)

A

|  | Bifidobacteriaceae | | | Bacteroidaceae | | | Pasteurellaceae | | |
| --- | --- | --- | --- | --- | --- | --- | --- | --- | --- |
| week | 0 | 4 | 8 | 0 | 4 | 8 | 0 | 4 | 8 |
| Mean (%) | 10.437 | 18.404 | 12.972 | 6.902 | 3.242 | 7.278 | 0.436 | 0.112 | 0.031 |
| S.D | 7.91 | 11.237 | 7.397 | 8.419 | 6.933 | 11.724 | 1.187 | 0.332 | 0.065 |
| S.E | 2.19 | 3.12 | 2.05 | 2.34 | 1.92 | 3.25 | 0.33 | 0.09 | 0.02 |
| P value |  | 0.03318 | 0.60306 |  | 0.00236 | 0.60306 |  | 0.3125 | 0.00298 |

B

|  | Bifidobacterium | | | Bacteroides | | | Holdemanella | | | Haemophilus | | |
| --- | --- | --- | --- | --- | --- | --- | --- | --- | --- | --- | --- | --- |
| week | 0 | 4 | 8 | 0 | 4 | 8 | 0 | 4 | 8 | 0 | 4 | 8 |
| Mean (%) | 10.434 | 18.395 | 12.97 | 6.902 | 3.242 | 7.278 | 0.617 | 1.018 | 0.265 | 0.432 | 0.11 | 0.031 |
| S.D | 7.908 | 11.236 | 7.396 | 8.419 | 6.933 | 11.724 | 1.359 | 2.874 | 0.559 | 1.179 | 0.328 | 0.065 |
| S.E | 2.19 | 3.12 | 2.05 | 2.34 | 1.92 | 3.25 | 0.38 | 0.8 | 0.16 | 0.33 | 0.09 | 0.02 |
| P value |  | 0.03318 | 0.60306 |  | 0.00236 | 0.60306 |  | 0.21498 | 0.02852 |  | 0.34722 | 0.00374 |
