# Supplementary material for: Regulatory effects of Lactobacillus plantarum HY7714 on skin health by improving intestinal condition
Source: PLoS One. 2020 Apr 10;15(4):e0231268. doi: 10.1371/journal.pone.0231268 (PMC7147770; doi:10.1371/journal.pone.0231268)
Supplement: S2 Table — Zonulin (A), Calprotectin (B), MMP-2 (C), and MMP-9 (D). (DOCX) [file pone.0231268.s004.docx]

**S2 Table. Biomarker concentrations in plasma before and after HY7714 consumption** Zonulin(A), Calprotectin(B), MMP-2(C), and MMP-9(D)

A

|  | Zonulin | | |
| --- | --- | --- | --- |
| week | 0 | 4 | 8 |
| mean(ng/ml) | 26.40 | 18.72 | 19.22 |
| S.D | 10.84 | 5.74 | 5.22 |
| S.E | 3.01 | 1.59 | 1.45 |
| P value |  | 0.0193 | 0.0088 |

B

|  | Calprotectin | | |
| --- | --- | --- | --- |
| week | 0 | 4 | 8 |
| mean(ng/ml) | 23.66 | 17.42 | 17.41 |
| S.D | 8.08 | 4.57 | 6.9 |
| S.E | 2.24 | 1.27 | 1.91 |
| P value |  | 0.0131 | 0.0232 |

C

|  | MMP-2 | | |
| --- | --- | --- | --- |
| week | 0 | 4 | 8 |
| mean(ng/ml) | 263 | 163 | 88 |
| S.D | 137.23 | 28.47 | 26.34 |
| S.E | 38.06 | 7.9 | 7.31 |
| P value |  | 0.0015 | 0.0015 |

D

|  | MMP-9 | | |
| --- | --- | --- | --- |
| week | 0 | 4 | 8 |
| mean(ng/ml) | 252 | 232 | 114 |
| S.D | 153.1 | 105.04 | 93.12 |
| S.E | 42.46 | 29.13 | 25.83 |
| P value |  | 0.7039 | 0.0071 |
